# Comparing the Effectiveness of Individual Coaching, Self-Coaching, and Group Training: How Leadership Makes the Difference

**DOI:** 10.3389/fpsyg.2016.00629

**Published:** 2016-05-03

**Authors:** Sabine Losch, Eva Traut-Mattausch, Maximilian D. Mühlberger, Eva Jonas

**Affiliations:** ^1^Division of Economic and Organizational Psychology, Department of Psychology, University of SalzburgSalzburg, Austria; ^2^Division of Social Psychology, Department of Psychology, University of SalzburgSalzburg, Austria

**Keywords:** coaching, training, effectiveness, procrastination, transformational leadership, transactional leadership, autonomy support, intrinsic motivation

## Abstract

Few empirical studies have used a randomized controlled design to evaluate the impact of coaching, and there are even fewer that have compared coaching with other interventions. In the current field study, we investigated the relative effectiveness of coaching as an intervention to reduce procrastination. In a randomized controlled study, participants (*N* = 84) were assigned to an individual coaching, a self-coaching, a group training, or a control group condition. Results indicate that individual coaching and group training were effective in reducing procrastination and facilitating goal attainment. Individual coaching created a high degree of satisfaction and was superior in helping participants attaining their goals, whereas group training successfully promoted the acquisition of relevant knowledge. The results for the self-coaching condition show that independently performing exercises without being supported by a coach is not sufficient for high goal attainment. Moreover, mediation analysis show that a coach’s transformational and transactional leadership behavior influenced participants’ perceived autonomy support and intrinsic motivation, resulting in beneficial coaching outcomes. The results may guide the selection of appropriate human resource development methods: If there is a general need to systematically prepare employees to perform on specific tasks, group training seems appropriate due to lower costs. However, when certain aspects of working conditions or individual development goals are paramount, coaching might be indicated. However, further research is needed to compare the relative effectiveness of coaching with other interventions in different contexts.

## Introduction

In today’s fast-changing economy, the growth, productivity, and continuity of an organization are determined by employees’ professional and personal qualifications (Kauffeld, 2010; Salas et al., 2012). Consequently, the demand for increasing employees’ skills, knowledge, and productivity is high. One of the most widely used methods for enhancing individual and organizational performance is training (Arthur et al., 2003). The Industry Report (Training, 2014) stated that U.S. companies with 100 or more employees invested $62 billion in training in 2014. Indeed, there is a large body of literature on the effectiveness of organizational training suggesting that this investment is justified (e.g., Morris and Robie, 2001; Arthur et al., 2003; Keith and Frese, 2008; Salas et al., 2008). Over the past decade, however, organizations have increasingly relied on workplace and executive coaching, which has grown into a mainstream developmental activity (Grant et al., 2010).

According to the International Coach Federation (ICF)^1^, a total of 2,100 professional coaches were operating globally in 1999. By 2012 there were 47,500. In North America that year, for example, the total annual revenue from coaching was said to be $707 million. Yet, in terms of the impact of coaching on organizations, the picture is less clear than what is known about training outcomes. Although an abundance of coaching literature exists, the majority of the published empirical papers consist of contextual or survey-based research, giving useful information about, for instance, the delivery of coaching services rather than about coaching effectiveness (Grant, 2013a). Two quantitative reviews have summarized the research on the effectiveness of coaching. In one meta-analysis, De Meuse et al. (2009) examined executive coaching outcomes by estimates of return on investment. In another, Theeboom et al. (2014) shed light on the beneficial individual-level outcomes of coaching, such as performance or skills, well-being, coping, work attitudes, and goal-directed self-regulation. These reviews show how coaching affects individual and organizational development. However, as empirical evidence is still scarce and the literature provides mixed results, there remains reasonable doubt if the individual and organizational benefits of coaching can outweigh its high costs (Leonard-Cross, 2010). Nevertheless, although contemporary coaching research is in its infancy, coaching practice is gaining ground (Grant et al., 2010).

Overall, the figures presented demonstrate the willingness of organizations to spend vast sums of money on personnel and executive development (Bozer and Sarros, 2012). But how does one know what method is appropriate to attain certain organizational and personal goals? The literature provides some comparative research on different coaching approaches and methods. For example, in one study, researchers examined the relative effectiveness of external coaching, peer coaching, and self-coaching for improving the performance of participants in two master of business administration programs (Sue-Chan and Latham, 2004). External coaching and self-coaching were more effective for improving students’ interpersonal team-playing skills and course grades than peer coaching, and external coaching was most effective in enhancing performance and satisfaction. Another study compared professional and peer life coaching and found that professional coaching was more effective than peer coaching or no coaching (control group) for enhancing engagement in the coaching process, goal commitment and goal attainment (Spence and Grant, 2007). To the best of our knowledge, to date there is little or no empirical research exploring the effectiveness of coaching in comparison to the more well established practice of training.

Therefore, we sought to explore the relative effectiveness of coaching compared to other forms of personnel and executive development, such as training and education interventions. The differentiation of coaching and the more established methods seems to be crucial for several reasons. First, although the body of literature on the effectiveness of coaching has been rapidly increasing in recent years, only a few empirical studies have used a randomized controlled design to evaluate the impact of coaching (Grant et al., 2010). Drawing on a solid theoretical framework, our empirical investigation of coaching effectiveness further enhances the quality of coaching outcome research and contributes evidence-based results (Cavanagh et al., 2005). Second, the comparative evaluation of coaching effectiveness not only answers the question if coaching is effective, but also may provide evidence of the inherent beneficial effects and limitations of the methods compared.

### Functional and Structural Differentiation of Coaching and Training

Coaching can be defined as a collaborative helping relationship, where coach and client (“coachee”) engage in a systematic process of setting goals and developing solutions with the aim of facilitating goal attainment, self-directed learning, and personal growth of the coachee (Grant and Stober, 2006; Grant, 2013b). The coachee’s responsibility is to implement action steps to achieve defined goals, while the coach keeps the coachee on track by managing the complex goal attainment process (Grant, 2013b). The coach’s function includes making explicit the difference between coaching and other forms of interventions (e.g., psychotherapy or expert counseling), setting clear agreements, clarifying respective responsibilities, and co-creating a supportive working relationship, as well as eliciting a thought-provoking and creative process through active listening and challenging questions (Grant, 2013b; “International Coach Federation,” 2015)^2^ In contrast, traditional training is a planned and systematic process that promotes the acquisition of relevant knowledge, skills, and attitudes through instruction, demonstration, practice, and immediate feedback about trainees’ performance (Salas and Cannon-Bowers, 2001; Salas et al., 2012). From these descriptions, significant differences regarding the role of coach and trainer during the process become apparent: A trainer follows a predetermined agenda and structure, providing instructions for achieving performance on relevant job or task requirements (Grant, 2001; Salas et al., 2012). In contrast, a coach follows the coachee’s agenda, providing support to create individual solutions tailored to the coachee’s specific needs (Grant, 2001). Therefore, coaching should be more effective in enhancing a clients’ work-related performance. In line with recent research finding that one of a coach’s core competencies is the ability to lead the client through the coaching process (Mühlberger and Traut-Mattausch, 2015) we argue that a coach’s leadership behavior is a crucial factor for coaching success.

As Kemp (2009) has stated, the interaction of coach and coachee is similar to the relationship between a leader and an employee, with the aim of the coach or leader being to facilitate and guide the follower’s development and performance. Accordingly, there are significant overlaps between coaching and leadership (Mühlberger and Traut-Mattausch, 2015): Both can be seen as an interaction process where a coach/leader asserts influence on the achievement of goals (Von Rosenstiel, 2006), the creation of solutions, and the development of the motivation and competencies of their clients/followers (Grant and Stober, 2006; Bass and Bass, 2008). However, there are also differences. In the leader–follower relationship, the leader is in a hierarchically higher position than his or her followers and supports them in attaining organizational goals. In contrast, the coaching relationship is one of equals (Rauen and Eversmann, 2014) and the coach helps the coachee attain his or her personal goals (Mühlberger and Traut-Mattausch, 2015).

Mühlberger and Traut-Mattausch (2015) suggested applying the concept of transactional and transformational leadership (Bass and Avolio, 1993; Bass, 1998) in the context of coaching. One particular transactional leadership strategy—the contingent reward component, characterized by setting clear basic expectations and goals (Felfe, 2006)—is part of the coach’s role. For example, a coach shows contingent reward behavior by communicating that the coachee is responsible for the implementation of action steps and goal attainment (Mühlberger and Traut-Mattausch, 2015). There are also certain overlaps between coaching and transformational leadership in practices that facilitate personal growth and motivate followers to perform beyond expectations (Bass, 1999). Some of the strategies transformational leaders use—individualized consideration, intellectual stimulation, and inspirational motivation—correspond to characteristic coaching behaviors. Coaches show individualized consideration by acknowledging the needs and goals of the coachees and supporting his or her personal strengths. Coaches provide intellectual stimulation by encouraging the coachees to consider issues from new perspectives and by doing so, they challenge the coachees assumptions and ideas. Finally, coaches provide inspirational motivation by helping the coachees create an optimistic vision for their future (Mühlberger and Traut-Mattausch, 2015).

Recent research demonstrated that transactional and transformational leadership behavior can indeed be transferred to the coaching context (Mühlberger and Traut-Mattausch, 2015). In an experimental study, undergraduates who obtained group or dyadic coaching to reduce procrastination behavior had higher goal attainment, goal commitment and goal self-efficacy compared to a control group. Undergraduates in the dyadic coaching setting, compared to those in the group coaching setting, showed higher increases in goal attainment, intrinsic motivation, and goal-related self-reflection. These effects were mediated by transactional and transformational coaching behavior. However, group and dyadic coaching consisted of only one session lasting 1 h. To our knowledge, no research has so far examined transactional and transformational leadership behavior in a real coaching setting.

Furthermore, leadership research focuses on investigating the mechanisms by which effective leaders exert influence on followers’ behaviors and performance (e.g., Kark et al., 2003, 2015; Piccolo and Colquitt, 2006). When investigating the role of the coach as leader and therefore highlighting transactional and transformational coaching behaviors, it is consequently important to gain insight into how these coaching behaviors affect coachees’ behavior and performance. So far, however, it remains unclear how coaches’ leadership behavior is linked to coaching success. A further aim of the current study was, therefore, to extend the research on coachs’ transactional and transformational leadership behavior as the underlying mechanism for coaching effectiveness.

### The Present Study

We conducted a field experiment to examine the relative effectiveness of coaching. For this purpose, we designed several interventions aimed at reducing procrastination, as this topic is relevant in academic and organizational contexts. Procrastination is defined as “the voluntary delay of an intended and necessary and/or [personally] important activity, despite expecting potential negative consequences that outweigh the positive consequences of the delay (Klingsieck, 2013).” In one study, up to 70% of students stated that they procrastinate (Schouwenburg et al., 2004), and another study reported that 50% procrastinate chronically, causing severe consequences (Day et al., 2000). Even in the general population, procrastination is a common phenomenon with prevalence rates of 20 to 25% (Ferrari et al., 2007). To overcome procrastination and time management problems in academic or workplace settings, training (Claessens et al., 2007) and coaching (e.g., Karas and Spada, 2009; Schmidt and Thamm, 2008, Unpublished) interventions are commonly used. There also exist a great number of self-help publications that give advice on how to cope with this problem (e.g., Ellis and Knaus, 1977; Fiore, 2007; Neenan and Dryden, 2013; Taylor, 2014).

This raises the question if following written instructions and doing exercises without being supported by a coach is sufficient to gain a benefit. To investigate which method is more effective and why, we compared individual coaching with self-coaching, group training, and no intervention (control group). Following Kirkpatrick’s (1959/1994) four-level model of training and learning evaluation, we asked participants to complete multiple measures to assess the interventions’ impact on satisfaction (*reaction*), content-related knowledge (*learning*), goal attainment, and state procrastination (*behavior*). Considering the characteristic features of individual coaching, self-coaching, and group training, we hypothesized that there would be a difference in the effectiveness of interventions on the different evaluation criteria.

The *reaction* criteria represent participants’ affective and attitudinal responses to the intervention (Arthur et al., 2003). Research suggests that clients experience the interaction with a coach providing feedback and support as important and beneficial, leading to higher client satisfaction ratings as opposed to those in self-coaching (Offermanns, 2004; Sue-Chan and Latham, 2004). As training is also characterized by immediate feedback between a trainer and clients (Salas and Cannon-Bowers, 2001; Salas et al., 2012) or by support trainees receive from their peers (Colquitt et al., 2000), we would assume similar beneficial effects on clients’ satisfaction ratings for training. Therefore we expected that the level of satisfaction among participants in the three intervention groups (individual coaching, self-coaching, group training) would be higher than among participants in the control group (Hypothesis 1.1). However, we expected that the support and feedback of a coach or trainer would lead to a higher level of satisfaction among participants in the individual coaching and group training conditions than among participants in the self-coaching condition (Hypothesis 1.2.). The *learning* criteria were the learning outcomes of the intervention that are usually assessed by paper-and-pencil tests (Arthur et al., 2003). Unlike for individual coaching or self-coaching, training is content based rather than process based (Clegg et al., 2003). Thus, a main component of the group training condition was the imparting of relevant knowledge on procrastination, the mechanisms of self-control and motivation, and time management techniques, helping participants overcome procrastination. We therefore hypothesized that participants in the group training condition would score higher on a multiple choice test on content-related knowledge than participants in the individual coaching, self-coaching, and control group conditions (Hypothesis 2). The *behavioral* criteria were the effects of the intervention on participants’ actual performance (Arthur et al., 2003). In the three intervention groups (individual coaching, self-coaching, group training), participants set goals and performed the same self-reflection and action-planning tasks designed to reduce procrastination. We hypothesized that participants in the three intervention groups would have higher goal attainment than participants in the control group (Hypothesis 3.1). For the tasks performed, the trainer provided instructions and exercises for overcoming procrastination, whereas the role of the coach was to monitor the coachees’ goal-attainment process and help them create individual solutions. Some important differences between the individual coaching and group training setting becomes apparent here: first, the focus on the goal attainment process is a core component of coaching conversations (Grant, 2012) and there is evidence that coaching increases goal attainment and performance (e.g., Grant, 2003, 2014; Green et al., 2006; Ianiro et al., 2013). Second, the problem-solving process characteristic for coaching involves helping the coachees to reflect on their thoughts, feelings, and behaviors which is associated with the facilitation of coachees’ metacognitive skills (Grant, 2001). Metacognitive skills have been shown to be crucial to improving task-relevant performance (Zimmermann and Schunk, 2011). Third, group settings like the traditional training entail the advantage of shared experiences and support from peers (Nicholas and Twaddell, 2008) when performing exercises to enhance specific skills. In contrast, however, individual coaching is a dyadic helping relationship (Jowett et al., 2012) that enables more individualized support and attention from the coach to meet the very specific developmental needs of the client (Rauen, 2005; Nicholas and Twaddell, 2008). With regard to the self-coaching condition, participants did not receive the support of a coach or from peers. Thus, we assumed that the success of the intervention would highly depend on participants’ self-leadership skills, that is, on their cognitive and behavioral strategies to motivate and direct themselves toward desired outcomes (Manz and Neck, 2002). Consequently, we expected that participants in the individual coaching would have higher goal attainment than participants in the self-coaching or group training condition (Hypothesis 3.2) and participants in the group training condition would have higher goal attainment than participants in the self-coaching condition (Hypothesis 3.3). In accordance with the hypotheses on goal attainment, we hypothesized that participants in the intervention groups (individual coaching, self-coaching, group training) would reduce procrastination more than participants in the control group (Hypothesis 4.1). However, we expected participants in the individual coaching condition to reduce procrastination more than participants in the self-coaching or group training condition (Hypothesis 4.2) and participants in the group training condition to reduce procrastination more than participants in the self-coaching condition (Hypothesis 4.3).

**FIGURE 1:**
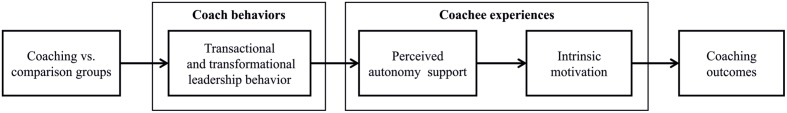
**Proposed theoretical model illustrating that the influence of a coach’s leadership behavior on a coachee’s experiences (perceived autonomy support and intrinsic motivation) has an effect on coaching outcomes**.

According to empirical findings, we have already noted that coaches show transactional and transformational leadership behavior, and that these leadership behaviors are positively related to coaching effectiveness (Mühlberger and Traut-Mattausch, 2015). But how do coaches’ transactional and transformational leadership behaviors influence coaching success? Some evidence comes from research on the effects of leadership behaviors on followers’ values, beliefs, and attitudes, demonstrating that transformational leaders enhance followers’ intrinsic motivation (Charbonneau et al., 2001; Shin and Zhou, 2003; Gagné and Deci, 2005; Sun et al., 2012). According to self-determination theory (SDT, Gagné and Deci, 2005), there are basic psychological needs that underlie intrinsic motivation, such as the need to feel competent, autonomous and related. With respect to the working environment, the support of followers’ autonomy turned out to be most essential factor to affect intrinsic motivation and thus, increase positive outcomes (Gagné and Deci, 2005). Transformational leaders create an autonomy-supportive interpersonal climate by acknowledging followers’ perspective, providing choice, reflecting feelings, providing rationales for requested behaviors, and encouraging self-regulation (Baard et al., 2004), resulting in high levels of intrinsic motivation and learning (Charbonneau et al., 2001; Hetland et al., 2011; Conchie, 2013). It is obvious that the type of communication through which transformational leaders create a climate of autonomy is parallel to the communication applied by coaches in coaching conversations. Thus, we would expect similar effects for the coaching behaviors on clients’ experiences and, in turn, on the coaching success. Moreover, we have already mentioned that coaches also show transactional (contingent reward) leadership behaviors. Although leadership literature mainly placed emphasis on the link between transformational leadership and autonomous forms of motivation, we postulate that the same process by which leaders have their effects on followers also applies to transactional leadership behaviors in the context of coaching. We argue that the coachees’ autonomy may be supported in that the coach clearly states that even if he or she actively supports the coachee in finding own solutions to attain his or her personal goals, the responsibility for goal attainment is still left with the coachee (Schein, 2003; Rauen, 2005). To sum up, we state that coaches’ leadership behaviors and coaching effectiveness are closely related to coachees’ experiences of autonomy support and intrinsic motivation. Therefore, we hypothesized that the difference between individual coaching, self-coaching, and group training on the evaluation criteria would be mediated by the coach’s transactional and transformational leadership behaviors and the coachee’s perceived autonomy support and intrinsic motivation (Hypothesis 5). **Figure 1** presents our proposed theoretical model.

## Materials and Methods

### Design

In this field study, a one-factorial between-subjects design with four experimental conditions (intervention: individual coaching vs. self-coaching vs. group training vs. control) was used in order to obtain data on the evaluation criteria reaction, learning, and behavior (Kirkpatrick, 1959/1994) at pretreatment and posttreatment.

### Participants

Participants were students of the University of Salzburg who regularly procrastinate on academic tasks. They were recruited by flyers and online advertisement for an intervention program. Psychology students received credit in return for research participation. A total of 134 respondents were randomly assigned to individual coaching, self-coaching, group training or a control group. If participants were unable to attend the prearranged training dates due to other commitments, they were randomly assigned to one of the other conditions. Fifty (37%) cases were excluded due to quitting the study or missing data; the remaining 84 participants allocated to the four conditions as follows: 23 (27%) individual coaching, 13 (15%) self-coaching, 27 (32%) group training, and 21 (25%) control group. The participants (20 men and 64 women) varied in age from 19 to 56 years. The average age of the sample was 25.95 years (*SD* = 7.11).

### Procedure

**FIGURE 2:**
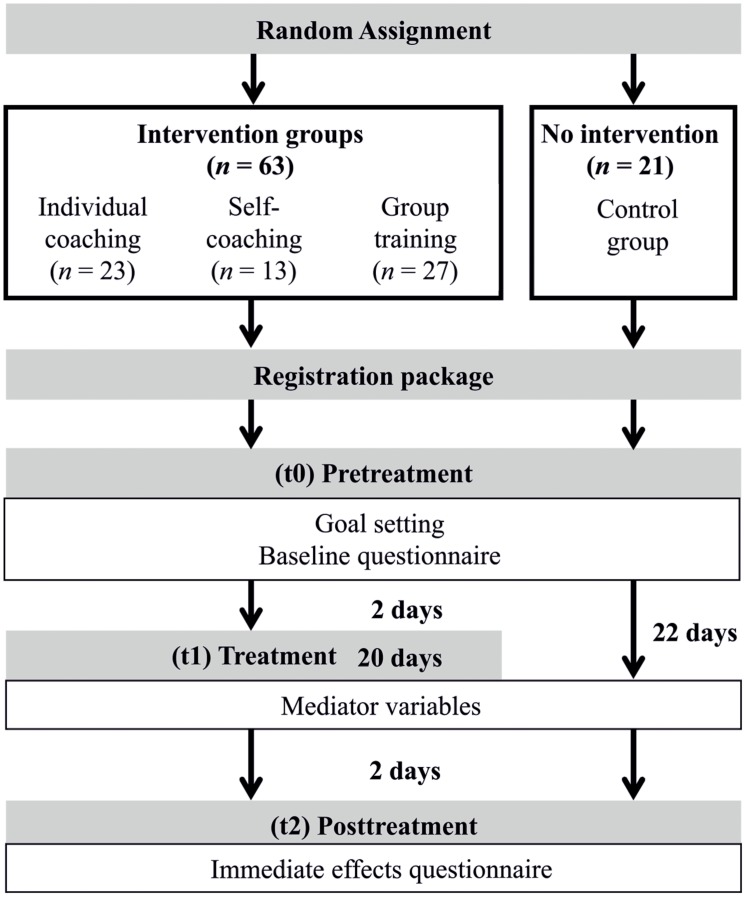
**Study procedure with time of assessment and the number of participants in the various conditions**.

**Figure 2** presents the study design. It includes information on the procedure and the size of the various conditions. The study was conducted at the Department of Psychology at the University of Salzburg. After the registration deadline and the assignment procedure, every participant received a registration package, which included an information letter regarding the specific intervention (only participants assigned to individual coaching, self-coaching, group training), a registration form, and an informed consent for participation and data collection. Prior to the interventions (t0), all participants were asked to identify individual goals they wanted to achieve relating to their procrastination and learning behavior. Then an online questionnaire was used to assess demographics and gather baseline data about participants’ individual goals and procrastination. Participants in the intervention groups completed online evaluation questionnaires to assess mediator variables in the course of the intervention (t1) and the immediate effects of the intervention at the end of the intervention program (t2).^3^ By contrast, after the assessment of baseline data at (t0), participants in the control group did not receive any further intervention; they only answered online evaluation questionnaires that were presented in parallel to the intervention group surveys. After data collection had been finished, participants in the control group were offered the self-coaching intervention to support attainment of their goals.^4^

### Intervention Groups

The individual coaching, self-coaching, and group training conditions consisted of three sessions of approximately 2 h each with 10 days in between. Session 1 focused on the discrepancy between an actual and desired state and setting personal goals. Session 2 addressed the identification of dysfunctional cognitions and patterns of behavior as well as the development of useful strategies. Session 3 aimed at working out an action plan and considering potential obstacles to goal attainment. Optionally, all participants were offered a follow-up meeting to reflect on their progress and difficulties regarding the implementation of the action plan. To avoid a bias in data collection due to benefits resulting from the follow-up meeting, appointments were arranged after participants had completed the last questionnaire.

Coaches and trainers were blind regarding the study hypotheses and investigated variables. We used guidelines to instruct coaches and trainers about the content and procedures of the individual units. Coaches were eight master’s students in psychology who had successfully completed a professional 1-year education program (for education concept see Braumandl et al., 2013). Trainers were three master students in psychology that were involved in designing the training program.

#### Individual Coaching

The individual coaching was characterized by systematic support of the coachee in setting appropriate goals, reflecting on the progress toward the attainment of these goals, and developing an individual approach that related to the client’s personal goals. The first coaching session proceeded as follows: (1) introduction of coach and coachee, (2) clarification of coaching relationship, (3) support of the coachee during goal setting, and (4) receipt of the first take-home exercise. The aim of the exercise was to identify cognitions and behavior patterns associated with procrastination situations. The second coaching session included (1) a review of the previous session and (2) a discussion of the first take-home exercise, both focusing on the coachee’s goals, and (3) receipt of the second take-home exercise. The aim of the exercise was to work out an individualized action plan. The third coaching session included (1) a review of the previous session and (2) a discussion of the second take-home exercise, both focusing on the coachee’s goals, and (3) coachee’s feedback on the coaching process. At the beginning and end of each session, the coachee rated his or her progress toward goal attainment.

#### Self-coaching

The structure and materials used in the self-coaching condition were equivalent to those used for individual coaching, except the role of coaches was replaced by written instructions in the form of a self-coaching manual. Participants received the self-coaching units by email and were told to return the completed units to the sender (i.e., the contact person). It was up to the participants themselves to set goals, reflect on the exercises, and identify the relevance of the insights for their personal goals.

#### Group Training

The group training intervention was designed as a standard training commonly employed in business. Therefore, the structure was equivalent to individual coaching, but the methods and materials had to be applicable to a group setting. Training was characterized by the systematic acquisition of knowledge and skills that relate to the reduction of procrastination. The first training session proceeded as follows: (1) introduction of trainer and trainees, (2) theoretical explanation on the phenomenology of procrastination following an example case, (3) practice in identifying dysfunctional cognitions and behaviors, and (4) theoretical input and practice in goal setting. The second training session included (1) a review of the previous session, (2) theoretical input and practice in replacing the dysfunctional cognitions and behaviors identified in the previous session with functional cognitions and behaviors, (3) theoretical input on the self-control mechanism, time management techniques and motivation, and (4) practice in using immediate gratification to maintain motivation. The third included (1) a review of the previous session, (2) an explanation of obstacles that can impede goal attainment and (3) practice in the use of time management techniques presented in the previous session to deal with these obstacles, (4) explanation of and practice in filling out an action plan, and (5) rating of goal attainment and trainees’ feedback on the training.

### Measures^5^

#### Effectiveness Measures

**Satisfaction** was assessed with the Coaching-Outcome-Short Scale (Schmidt and Thamm, 2008, Unpublished). Six items were adapted to measure participants’ satisfaction with the coach, contact person, or trainer^6^ (“How satisfied were you with your coach/contact person/trainer?”), the intervention program (“How satisfied were you with the coaching/self-coaching/training/goal setting?”), goal attainment (“How satisfied are you with the progress toward achieving your goals?”), outcome (“How satisfied are you in general with the coaching/self-coaching/training outcome?”), personal change (“How satisfied are you with the personal change through coaching/self-coaching/training/goal setting?”), and level of self-satisfaction (“How satisfied were you with yourself in the coaching/self-coaching/training/goal attainment process?”). Items were rated on a 10-point scale ranging from 1 (*not at all satisfied*) to 10 (*very satisfied*). Satisfaction was measured at t2 (α = 0.90).

A multiple-choice test containing 14 questions (e.g., “Which statement about procrastination is true?”) was created to check participants’ acquired knowledge regarding procrastination, the mechanisms of self-control and motivation, and time management techniques. Each item had four answers with between one and four correct alternatives. Participants got one point for every item answered completely right. They got no points for partially or completely incorrect items. Acquired **content-related knowledge** was assessed at t2.

**Goal attainment** was assessed on a process evaluation scale (Braumandl and Dirscherl, 2005; Ianiro et al., 2014; Biberacher, 2010, Unpublished), ranging from 1 (*not at all achieved*) to 10 (*fully achieved*). Participants rated their degree of actual goal attainment (“As of right now, to what extent have you attained this goal?”) for up to three goals. Goal attainment was assessed at t0 and t2.

To assess state **procrastination**, we used a 12-item subscale of the Academic Procrastination State Inventory (APSI, Schouwenburg, 1995; German translation by Helmke and Schrader, 2000). The subscale measures state procrastination in terms of delay, concentration deficits, and lack of energy. Participants had to rate how frequently in the last week they had engaged in procrastination-related thoughts and behaviors (e.g., “Put off the completion of a task”) on a 5-point scale ranging from 0 (*never*) to 4 (*always*). Procrastination was measured at t0 (α = 0.75) and t2 (α = 0.89).

#### Mediator Variables

The following mediators were measured during the intervention program (t1) between pre- and posttreatment^7^: **Transactional and transformational leadership behaviors** of the coach, trainer, or contact person were measured with an adapted version of the Multifactor Leadership Questionnaire (MLQ, Bass and Avolio, 1995; German translation by Felfe and Goihl, 2002). Transformational leadership behaviors (α = 0.93, 12 items, e.g., “The coach helped me find new ways to reach my goals”) and the transactional leadership behavior contingent reward (α = 0.83, 4 items, e.g., “The coach made it clear that I am responsible for attaining my goals”) were measured on a 5-point scale ranging from 1 (*not at all*) to 5 (*frequently*).

To assess participants’ perceptions of the degree to which their coach, trainer, or contact person supported **autonomy**, we used an adapted version of the Work Climate Questionnaire (WCQ, Baard et al., 2004). Participants answered 15 items (α = 0.90, e.g., “My coach listens to how I would like to do things”) on a 7-point scale ranging from 1 (*not at all true*) to 7 (*very true*). Items were translated into German and back-translated into English by a native speaker to ensure accurate interpretation.

The degree of participants’ perceived **intrinsic motivation** was assessed with a short intrinsic motivation scale (Kurzskala Intrinsischer Motivation, KIM, Wilde et al., 2009). Participants rated nine items (α = 0.91, e.g., “I think that working on my learning behavior is very interesting”) on a 5-point scale from 1 (*not at all*) to 5 (*very much*).

## Results

### Effectiveness of Interventions

To test the hypotheses on the effectiveness of individual coaching, self-coaching, and group training, the intervention programs were evaluated by comparing the degree of content-related knowledge and satisfaction at posttreatment (t2). To test for changes in goal attainment progress and procrastination, we calculated the difference between each participant’s rating of goal attainment and procrastination at posttreatment (t2) and pretreatment (t0). Significant main effects were followed up by post hoc comparisons using Fisher’s least significant difference test. **Table 1** presents the means and standard deviations of the dependent variables at pretreatment (t0) and posttreatment (t2).

**Table 1:** Means and Standard Deviations for the effectiveness measures (Time 0, Time 2) and mediator variables (Time 1).

Study variable	Condition							
	
	Individual coaching		Self-coaching		Group training		Control group	
				
	*M*	*SD*	*M*	*SD*	*M*	*SD*	*M*	*SD*
**Time 0 variables**								
Goal attainment	2.91	1.44	3.15	1.57	2.81	1.42	3.19	2.06
Procrastination	2.70	0.46	2.70	0.38	2.57	0.46	2.65	0.68
**Time 1 variables**								
TFL	4.15	0.55	3.08	1.00	3.54	0.76	–	–
TAL	3.97	0.63	3.45	0.82	3.66	0.83	–	–
PAS	5.99	0.54	5.09	0.99	5.48	0.98	–	–
IMOT	3.90	0.56	3.26	0.67	3.55	0.59	2.85	0.91
**Time 2 variables**								
Satisfaction	7.52	1.33	6.21	1.75	6.59	1.79	5.23	2.08
Content-related knowledge	2.90	1.29	2.75	2.17	4.30	1.65	–	–
Goal attainment	6.96	1.49	5.15	2.08	6.07	2.24	5.62	2.22
Procrastination	1.89	0.61	2.15	0.71	1.99	0.63	2.42	0.83


*TFL, transformational leadership behavior; TAL, transactional leadership behavior; PAS, perceived autonomy support; IMOT, intrinsic motivation.*

#### Preliminary Analyses

First, we examined our data for potential differences in the baseline measurements. One-way analyses of variance (ANOVAs) showed that the four groups were equivalent at baseline on actual state of goal attainment for the main goal, *F*(3,80) < 1, *p* = 0.847, η^2^ = 0.01, and procrastination, *F*(3,80) < 1, *p* = 0.806, η^2^ = 0.01.

#### Satisfaction

Hypothesis 1.1 stated that the level of satisfaction among participants in the intervention groups would be higher than among participants in the control group. A one-way ANOVA revealed a significant effect of intervention on satisfaction, *F*(3,80) = 6.37, *p* = 0.001, η^2^ = 0.019. *Post hoc* analyses indicated that participants’ satisfaction was significantly higher for individual coaching (*M* = 7.52, *SD* = 1.33), *p* < 0.001, and group training (*M* = 6.59, *SD* = 1.79), *p* = 0.009, compared to the control group (*M* = 5.23, *SD* = 2.08), whereas self-coaching (*M* = 6.21, *SD* = 1.75) did not significantly differ from the control group (*p* = 0.117). Thus, Hypothesis 1.1 is partly supported. Hypothesis 1.2 posited that the level of satisfaction would be higher among participants in the individual coaching and group training conditions than among participants in the self-coaching condition. Participants’ satisfaction for individual coaching was marginally significantly higher than for group training (*p* = 0.065) and significantly higher compared to self-coaching (*p* = 0.035). However, group training did not significantly differ from self-coaching (*p* = 0.528), which partly supports Hypothesis 1.2.

#### Content-Related Knowledge

Hypothesis 2 posited that participants in the group training condition would achieve higher scores in a multiple-choice test regarding procrastination, self-control and motivational mechanisms, and time management techniques than participants in the individual coaching, self-coaching, and control group conditions. As only five multiple-choice tests were returned by the participants of the control group, their data could not be included in the analysis. In individual coaching, five cases were excluded due to missing values. A one-way ANOVA revealed a significant effect of intervention on content-related knowledge, *F*(2,56) = 5.59, *p* = 0.006, η^2^ = 0.17. *Post hoc* analysis indicated that scores achieved on the multiple-choice test were significantly higher for participants receiving group training (*M* = 4.30, *SD* = 1.65) compared to individual coaching (*M* = 2.90, *SD* = 1.29), *p* = 0.006, and self-coaching (*M* = 2.75, *SD* = 2.17), *p* = 0.010. There was no statistically significant difference between the individual coaching and self-coaching conditions in content-related knowledge (*p* = 0.806). These findings support Hypothesis 2.

#### Goal Attainment

Participants set up to three individual goals. Their first goal represented the main goal and was well documented in all conditions. As not all participants set a second or third goal or, especially in the self-coaching and control group, documented changes of second or third order goals from t0 to t2, we could not clearly match goal-attainment ratings. Hence, goal-attainment progress was analyzed only for the first goal.^8^ In Hypothesis 3.1 we stated that participants in the intervention groups would have higher goal attainment than participants in the control group. A one-way ANOVA revealed a significant effect of intervention on goal attainment for the main goal, *F*(3,80) = 2.79, *p* = 0.046, η^2^ = 0.10. *Post hoc* analysis indicated that goal attainment progress was significantly higher for the individual coaching condition (*M* = 4.04, *SD* = 1.74) compared to the control group (*M* = 2.43, *SD* = 2.46), *p* = 0.025. The self-coaching (*M* = 2.00, *SD* = 2.16, *p* = 0.607) and group training (*M* = 3.22, *SD* = 2.73, *p* = 0.228) conditions did not significantly differ from the control group, thus partly supporting Hypothesis 3.1. Hypothesis 3.2 predicted that participants in the individual coaching condition would have higher goal attainment than participants in the self-coaching or group training condition and Hypothesis 3.3 posited that participants in the group training condition would have higher goal attainment than participants in the self-coaching condition. The increase in goal attainment was higher for the individual coaching condition than for the self-coaching condition (*p* = 0.014), but the group training condition did not significantly differ from the individual coaching (*p* = 0.243) and self-coaching conditions (*p* = 0.116). Thus, hypothesis 3.2 is partly supported and Hypothesis 3.3 is not supported.

#### Procrastination

In Hypothesis 4.1, we predicted that participants in the intervention groups would reduce procrastination more than participants in the control group. A one-way ANOVA revealed a significant effect of intervention on procrastination, *F*(3,80) = 3.17, *p* = 0.029, η^2^ = 0.11. *Post hoc* analysis indicated that individual coaching (*M* = 0.80, *SD* = 0.60, *p* = 0.003) reduced procrastination significantly more than no intervention (control group; *M* = 0.23, *SD* = 0.72) and group training (*M* = 0.57, *SD* = 0.58, *p* = 0.060) reduced procrastination marginally significantly more than no intervention. The self-coaching group (*M* = 0.54, *SD* = 0.49) did not significantly differ from the control group in the reduction of procrastination (*p* = 0.155), which partly supports Hypothesis 4.1. Our next hypotheses stated that participants in the individual coaching condition would reduce procrastination more than participants in the self-coaching or training condition (Hypothesis 4.2) and participants in the group training condition would reduce procrastination more than participants in the self-coaching condition (Hypothesis 4.3). Individual coaching did not significantly differ from the self-coaching (*p* = 0.230) and group training conditions (*p* = 0.193), and there was no statistically significant difference between the group training and self-coaching condition (*p* = 0.888). These findings reject Hypotheses 4.2 and 4.3.

### Mediation Effects: Influence of Transactional and Transformational Leadership Behaviors on Coachees’ Experiences and Effectiveness

To test Hypothesis 5, that coaches’ transactional and transformational leadership behaviors would influence participants’ experiences and, in turn, that these experiences would explain the difference in the effectiveness of the intervention programs, we performed serial multiple mediation analyses with the software PROCESS (Hayes, 2013, Model 6). We used a 95% bias-corrected bootstrap confidence interval (95% BC CI) and 5,000 bootstrap samples. Transformational leadership behavior, transactional leadership behavior (contingent reward), perceived autonomy support, and intrinsic motivation were used as mediators. On the basis of previous results, we first employed Contrast A (individual coaching vs. self-coaching, group training vs. self-coaching as a covariate) as independent variable to examine the effects on the dependent variable goal attainment. Second, we employed Contrasts A and B (individual coaching vs. group training, self-coaching vs. group training as a covariate) as independent variables to examine the effects on the dependent variable satisfaction. Means and standard deviations of the mediator variables (t1 variables) are displayed in **Table 1.**

#### Goal Attainment

**FIGURE 3:**
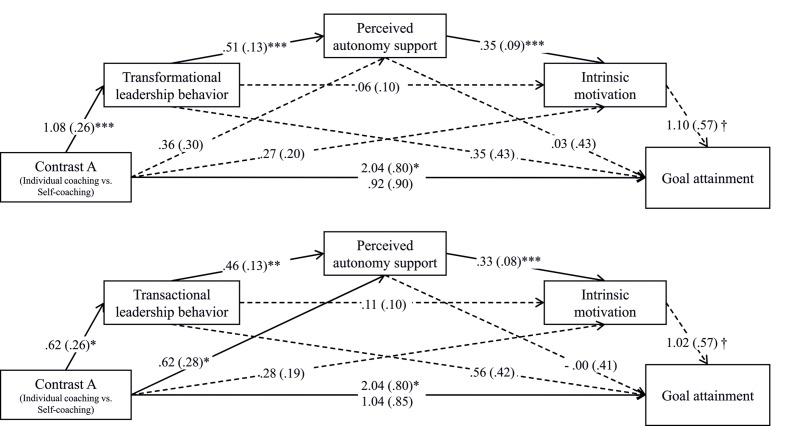
**The effect of intervention group on goal attainment via coaching behaviors (transformational or transactional leadership behavior) and coachee experiences (perceived autonomy support and intrinsic motivation).** ^†^*p* < 0.10, ^∗^*p* < 0.05, ^∗∗^*p* < 0.01, ^∗∗∗^*p* < 0.001.

Mediation analyses revealed a significant total effect of individual coaching on goal attainment for Contrast A, *b* = 2.04, *SE* = 0.80, *t*(60) = 2.55, *p* = 0.013. The effect was non-significant when the potential mediators transformational leadership behavior, perceived autonomy support, and intrinsic motivation were added to the prediction, *b* = 0.92, *SE* = 0.90, *t*(60) = 1.03, *p* = 0.308. For Contrast A, the bootstrapped indirect effect of individual coaching via transformational leadership behavior, perceived autonomy support, and intrinsic motivation was significant in a positive direction, *b* = 0.21, *SE* = 0.14, 95% BC CI [0.05, 0.72]. Moreover, the total effect of individual coaching on goal attainment for Contrast A was non-significant when the potential mediators transactional leadership behavior, perceived autonomy support, and intrinsic motivation were added to the prediction, *b* = 1.04, *SE* = 0.85, *t*(60) = 1.23, *p* = 0.225. For Contrast A, the bootstrapped indirect effect of individual coaching via transactional leadership behavior, perceived autonomy support, and intrinsic motivation was significant in a positive direction, *b* = 0.10, *SE* = 0.07, 95% BC CI [0.02, 0.35]. Furthermore, the indirect effect of individual coaching via perceived autonomy support and intrinsic motivation was significant as well, *b* = 0.21, *SE* = 0.15, 95% BC CI [0.02, 0.72] (for the path coefficients see **Figure 3**).

### Satisfaction

**FIGURE 4:**
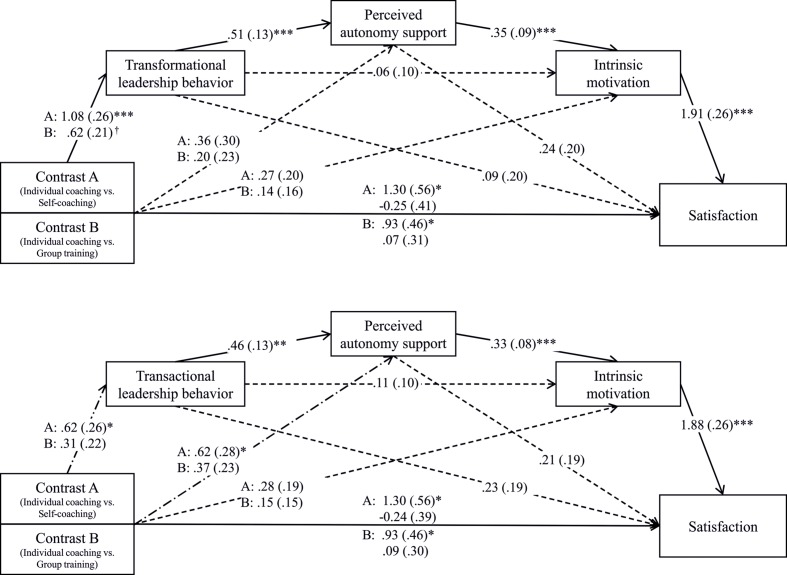
**The effect of intervention group on satisfaction via coaching behaviors (transformational or transactional leadership behavior) and coachee experiences (perceived autonomy support and intrinsic motivation).** ^†^*p* < 0.10, ^∗^*p* < 0.05, ^∗∗^*p* < 0.01, ^∗∗∗^*p* < 0.001.

Mediation analyses revealed a significant total effect of individual coaching on satisfaction for Contrast A, *b* = 1.30, *SE* = 0.56, *t*(60) = 2.31, *p* = 0.024, and Contrast B, *b* = 0.93, *SE* = 0.46, *t*(60) = 2.01, *p* = 0.049. The effects for both contrasts were non-significant when the potential mediators transformational leadership behavior, perceived autonomy support, and intrinsic motivation were added to the prediction, *b* = -0.25, *SE* = 0.41, *t*(60) = -0.60, *p* = 0.550 and *b* = 0.07, *SE* = 0.31, *t*(60) = 0.22, *p* = 0.827. For Contrasts A and B, the bootstrapped indirect effect of individual coaching via transformational leadership behavior, perceived autonomy support, and intrinsic motivation was significant in a positive direction, *b* = 0.36, *SE* = 0.16, 95% BC CI [0.16, 0.95] and *b* = 0.21, *SE* = 0.09, 95% BC CI [0.08, 0.49]. Moreover, the total effect of individual coaching on satisfaction was non-significant for both contrasts when the potential mediators transactional leadership behavior, perceived autonomy support, and intrinsic motivation were added to the prediction, *b* = -0.24, *SE* = 0.39, *t*(60) = -0.61, *p* = 0.550 and *b* = 0.09, *SE* = 0.30, *t*(60) = 0.28, *p* = 0.777. For Contrasts A and B, the bootstrapped indirect effect of individual coaching via transactional leadership behavior, perceived autonomy support, and intrinsic motivation was significant in a positive direction, *b* = 0.18, *SE* = 0.10, 95% BC CI [0.05, 0.50] and *b* = 0.09, *SE* = 0.06, 95% BC CI [0.00, 0.27]. Furthermore, the indirect effect of individual coaching via perceived autonomy support and intrinsic motivation was significant, as well, for Contrast A, *b* = 0.38, *SE* = 0.19, 95% BC CI [0.11, 0.87] and Contrast B, *b* = 0.23, *SE* = 0.14, 95% BC CI [0.02, 0.61] (for path coefficients see **Figure 4**).

In sum, the results, supporting Hypothesis 5, show that the higher goal attainment and satisfaction in the individual coaching compared to self-coaching and group training conditions were explained by the coaches’ behaviors and clients’ experiences. Clients experienced more transformational leadership behaviors in individual coaching that further led to the perception of autonomy support and intrinsic motivation, resulting in higher goal attainment and satisfaction. Likewise, the coaches’ transactional leadership behaviors contributed to clients’ experience of autonomy support and intrinsic motivation, resulting in higher goal attainment and satisfaction. Unlike transformational leadership behavior, however, coaches’ transactional leadership behavior was not necessarily required to influence clients’ experiences and in turn to explain differences in goal attainment and satisfaction.

## Discussion

In the current research we conducted a randomized controlled study to provide further evidence on the effectiveness of coaching in comparison to other interventions in the context of procrastination prevention. For this purpose, we created individual coaching, group training, and self-coaching interventions to assess their relative effectiveness as compared to a control group that received no intervention. Interventions were consistent regarding procedure and content, that is, participants set goals and performed the same self-reflection and action-planning tasks designed to reduce procrastination. However, the settings differed in respect to the specific intervention characteristics. Participants of the control group had to set goals with regard to their procrastination and learning behavior but did not receive any further treatment. Following Kirkpatrick’s (1959/1994) four-level model of training evaluation, we assessed participants’ self-rated satisfaction, content-related knowledge, goal attainment, and state procrastination as a measure of the effectiveness.

Contrary to our predictions, the increase in goal attainment in the group training condition did not significantly differ from the increase in the individual coaching, self-coaching, and control group conditions. That is, even if group training could help participants attain their goals, training was not more effective than merely setting a goal or engaging in goal-striving activities by oneself. As expected, individual coaching was the most effective intervention to facilitate participants’ goal attainment. Therefore, our findings support the understanding of coaching as goal-focused communication intended to help foster the regulation and direction of clients’ resources to create purposeful positive change (Grant and Stober, 2006; Grant, 2012). That participants of the control group reported some degree of goal-attainment progress was surprising, as they did not objectively receive any intervention. However, they set goals prior to data collection and answered effectiveness measures, as well. It is possible that the set goals indeed had an influence on the goal attainment of the control group. According to goal-setting theory (Locke and Latham, 2002), goals enhance performance by directing attention to goal-relevant cognitions and behaviors, increase motivation and persistence, and stimulate the regulation of task-related strategies (Wood and Locke, 1990). Another possible explanation is, that goal setting in the control group condition could have framed the perception of undergoing an intervention and, in turn, may have led participants to consider their goal-attainment progress with some pattern in mind, for example, that ratings must increase from the first to the second assessment. This is a characteristic of the “demand effect” that may particularly occur in within-subject designs (Gneezy, 2005; Charness et al., 2012). In light of this explanation, it is reasonable to question if the goal-attainment progress of participants in the control group was comparable to that of participants in the group training condition.

Moreover, participants in the individual coaching and group training conditions reported significantly less procrastination compared to those in the control group. Our findings show that both individual coaching and group training were effective interventions to reduce procrastination, which is in line with existing research on the effectiveness of procrastination and time-management-related interventions (e.g., Van Eerde, 2003; Green and Skinner, 2005; Karas and Spada, 2009; Schmidt and Thamm, 2008, Unpublished). Contrary to our assumption, however, individual coaching did not significantly differ from group training in the reduction of procrastination. As the training evaluation literature suggests a link between learning (e.g., declarative knowledge and skill acquisition) and behavioral change (Colquitt et al., 2000), our following results may shed light on this finding. As we assumed, participants who attended group training achieved higher scores in a multiple-choice test regarding procrastination, mechanisms of self-control and motivation, and time management techniques than participants in the individual coaching, self-coaching, and control group conditions. This result is not surprising, as one of the main objectives in group training was to impart knowledge and specific skills to overcome procrastination. In contrast, individual coaching focused on creating individual problem-solving strategies tailored to the respective goals and the needs of the participants. This involved supporting coachees in the planning, monitoring, and evaluation of goal-attainment strategies, which is associated with the development of metacognitive skills (Veenman and Elshout, 1999). Therefore, our findings point to different learning processes encouraged by individual coaching and group training: As metacognitive skills are procedural knowledge (Efklides, 2006), coaching primarily fostered a more implicit, self-directed learning process that may have enabled participants to successfully change their procrastination behavior. However, by enhancing relevant knowledge and skills (i.e., proper use of time-management techniques), group training primarily fostered a more explicit (declarative) learning process that may have enabled participants to apply the demonstrated solution strategies to their posttraining environment.

Another result was that participants in the self-coaching condition did not significantly differ in reducing procrastination from those in the individual coaching, group training, and control group conditions. Participants were able to improve their procrastination and learning behavior to some degree through self-coaching, though, they did not report less state procrastination compared to participants in the control group. Self-coaching is a self-directed learning and development process that is not supported by a coach; it requires very high self-regulation, self-motivation, and self-learning competencies (Lieser, 2012). As it is known that procrastination involves impaired self-regulation (Baumeister et al., 1994), this result is not surprising.

Regarding the reaction criteria, results revealed higher levels of satisfaction for participants in the individual coaching and group training conditions compared with the control group. However, satisfaction ratings among participants in the self-coaching condition were nearly equal to satisfaction ratings in the group training and control group conditions. Overall, satisfaction was highest in individual coaching, which even marginally differed from group training. In the current study, satisfaction ratings included information on, for example, how satisfied participants were with their goal-attainment progress or their personal change through the intervention. Hence, this difference (i.e., between individual coaching and group training) may have occurred because coaching specifically aims at systematically facilitating clients’ goal attainment process and personal growth (Grant and Stober, 2006), which is in line with our finding that coaching was most effective in supporting goal attainment.

We argued that the influence of a coach’s transactional and transformational leadership behavior on coachees’ experiences determines coaching success. The findings support Hypothesis 5: Coaches’ transactional and transformational leadership behaviors contributed to coachees’ experience of autonomy support and intrinsic motivation, resulting in higher goal attainment and satisfaction. Our results are in line with previous experimental research (Mühlberger and Traut-Mattausch, 2015) findings that coaches show transactional and transformational leadership behaviors when leading coachees through the process. We provide further evidence that individual coaching leads to more exposure of transactional and transformational leadership behavior compared with self-coaching and group training. Moreover, coaches’ transformational leadership behaviors were related to coachees’ perceived autonomy support. Thus it seems that coaching behaviors, for example, acknowledging coachees’ perspective and needs and involving coachees in the problem-solving process, are prerequisites to creating an autonomy-supportive environment. Transactional leadership behaviors (contingent reward), for instance, clarifying mutual expectations and responsibilities in the coaching agreement, were also positively related to coachees’ perceptions of autonomy support. Finally, coachees’ experience of autonomy gave rise to high levels of intrinsic motivation. According to self-determination theory, autonomy and intrinsic motivation are critical factors in behavior-change settings, producing behavioral outcomes that are more likely to succeed (Ryan and Deci, 2008; Ryan et al., 2011). In sum, these findings lend support to the idea that coaches’ transactional and transformational leadership behaviors are critical antecedents of coaching success. Furthermore, the results show that coaches’ leadership behaviors and coaching effectiveness are closely linked to coachees’ perceptions and motivation.

### Practical Implications

In general, the current study further contributes to the systematic evaluation of coaching effectiveness by drawing on the firm theoretical framework of transactional and transformational leadership research and using a randomized controlled design. This will be of benefit for coaching professionals, as they can refer to empirical, evidence-based results when offering coaching services. In this regard, the findings on the satisfaction with the interventions may be of particular relevance because clients can be considered customers (Allinger et al., 1997). Thus, when satisfaction with the provided services is high, there is a good chance for positive word-of-mouth communication (Allinger et al., 1997; Anderson, 1998). This may have an impact on the attendance, implementation, and subsequent funding of human resource development programs in organizations (Allinger et al., 1997). Moreover, in the service literature, word of mouth is regarded as a determinant of consumer choice and resulting competitive advantages in the marketplace (Chung and Darke, 2006) for professional coaches and trainers.

Our results on the relative effects on procrastination and goal attainment lend support to the view that individual coaching and group training are effective interventions for developing skills and changing behavior. This may help in the selection of the appropriate human resource development method for a given situation: To systematically prepare employees to perform on specific tasks (e.g., communication or time management skills), individual coaching and group training are similarly effective. As costs for group settings are lower than for dyadic settings (Mühlberger and Traut-Mattausch, 2015), group training should be considered as an intervention. However, individual coaching was superior in helping participants attain their goals. Therefore, coaching may be indicated when certain aspects of working conditions or individual development goals (e.g., intercultural communication skills or creating realistic timetables for a project) are paramount. Furthermore, when leading clients through the coaching process, coaches show transformational leadership behavior (Mühlberger and Traut-Mattausch, 2015). Transformational leadership is supposed to be effective under conditions that require leadership beyond the conferring of extrinsic reward and punishments—conditions that may involve unstable and non-routine situations in organizational change processes or resistance of followers (Shamir et al., 1993; Tonhäuser, 2013). Indeed, research demonstrated that executive coaching in times of organizational change was associated, for example, with executives’ and managers’ increased work-related goal attainment, enhanced solution-focused thinking, and a greater readiness and ability to deal with change (Grant, 2013b).

As our results of self-coaching suggest, independently performing exercises without being supported by a coach is not sufficient to enhance performance and facilitate goal attainment.

### Limitations and Future Research

One limitation of our study is that participants received a treatment of 6 h over a period of 3 weeks, where effectiveness was assessed immediately after the intervention. As procrastination is associated with severe self-regulatory failure (Baumeister et al., 1994), it would be useful to add further follow-up measurements to examine if the interventions were able to create sustainable change.

Second, it is to assume that our findings may be subject to common method bias, as effectiveness measures were limited to participants’ subjective self-assessments (Podsakoff et al., 2003). The reliance on self-report data is an important limitation in the current coaching evaluation literature (Ely et al., 2010). In particular, people tend to overestimate procrastination and performance when assessed using self-report instruments compared to externally assessed procrastination and performance (Rotenstein et al., 2009; Kim and Seo, 2015). Furthermore, this study was conducted in only one university. Therefore, findings from the present study might also be biased by the organizational culture of the university which may create an emotional climate both for employees and students (Giorgi, 2012, 2013), leading participants feeling obliged to give positive responses regarding expected study outcomes. To ensure valid results, objective criteria or multi-source data should be used to evaluate coaching outcomes (Ely et al., 2010). For example, we suggest investigating the relationship between procrastination and academic success by including external indicators of performance, such as final exam or course grades (Steel et al., 2001; Kennedy and Tuckman, 2013; Lowinger et al., 2014) or the proportion of scheduled exams actually passed as an effectiveness measure.

Third, we examined the effectiveness of interventions designed to reduce procrastination in an academic setting. Therefore, one might question if results can be extended to procrastination and time management issues in the workplace, and if the pattern of findings varies depending on the purpose of the interventions. More research is needed to investigate the relative effectiveness of coaching in, for example, managing conflicts or developing social and intercultural skills in the organizational context.

Fourth, the number of participants in each condition is very small due to the high dropout rate or missing data. In particular, there was a dropout of 20 (61%) cases in the self-coaching condition, followed by 17 (39%) in the group training, 9 (30%) in the control group, and 4 (15%) in the individual coaching condition. Therefore, results have to be considered with caution^9^ and further studies based on a larger sample size are required to confirm our findings and reach valid conclusions about the effects of self-coaching interventions (Nezu and Nezu, 2008). However, the dropout rates of the current research lead us to the assumption that the lacking interaction with a coach who keeps participants on track might be a problem inherent to self-coaching, making it difficult to examine the effects of this type of intervention.

Furthermore, the findings in the current research underline the characteristic benefits of interventions: Training was more effective in enhancing knowledge, whereas individual coaching particularly facilitated attainment of personal goals. It would be interesting to investigate the effectiveness of coaching when combined with group training. In one study, training followed by individual coaching sessions to work more on particular aspects that were personally relevant enhanced productivity more than training alone (Olivero et al., 1997). It remains to be examined if the effectiveness of the interventions combined goes beyond the effects of receiving only individual coaching. Moreover, individual coaching followed by group training could also be an effective strategy: through individual one-to-one coaching sessions, coaches can gain valuable insight into the nature of a given problem and related specific concerns of the clients. These information could consequently help to design training that is more accurately tailored to identified needs and the individual characteristics a client brings to the training environment — both factors that have significant influence on training success (Salas and Cannon-Bowers, 2001). Further research is needed to see how combined interventions are best designed to ensure the maximum effect of transfer.

In addition, like the majority of the outcome studies included in meta-analyses (De Meuse et al., 2009; Theeboom et al., 2014), we evaluated the effectiveness of coaching in terms of an intervention designed to modify specific, goal-relevant thoughts, attitudes, and behaviors. Besides, coaching aims at improving individuals’ functioning by enhancing their interpersonal and intrapersonal resources (Grant, 2012) which are known to serve preventive and protective functions (Di Fabio and Kenny, 2011; Di Fabio, 2015). For example, addressing a project manager’s abilities to cope with stressors at an early stage could help organizations to save costs related to poor time management, stress-related absenteeism, or burnout at a later stage (Duijts et al., 2008). Consequently, in line with findings on the preventive role of individual resources (Di Fabio and Kenny, 2011, 2012a,b, 2015; Di Fabio, 2015) and looking ahead to future coaching evaluation research, we emphasize to focus increasingly on coaching interventions from a preventive perspective.

Finally, we demonstrated that coaches’ transactional and transformational leadership behaviors increased perceived autonomy support and intrinsic motivation of the coachees, resulting in beneficial coaching outcomes. There is a need to investigate further the effects of coaches’ leadership behaviors on coachee perceptions. Leadership research has shown that transformational leadership behaviors increase trust in the leader (e.g., Podsakoff et al., 1990) and enhance perceived justice (e.g., Pillai et al., 1999). As psychotherapy research has shown, clients’ secure attachment to their therapist is important for changing symptom distress (De Haan, 2012; Wiseman and Tishby, 2014). Likewise, transformational leadership focuses on the development of subordinates to become autonomous and competent (Popper and Mayseless, 2003), emphasizing the role of transformational leaders as attachment figures (Davidovitz et al., 2007). It may be that transformational leadership behaviors make coaches appear trustworthy, promote the experience of justice, and give rise to coachees’ secure attachment to their coach.

## Conclusion

We examined the relative effectiveness of coaching in comparison with other interventions and provided evidence for the different impact they have on individual-level outcomes: our findings not only show that coaching and training effectively enhance performance, but also emphasize the beneficial effects of coaching on clients’ goal attainment. In addition, to shed light on the underlying mechanisms of coaching effectiveness, we extended recent research on coaches’ transactional and transformational leadership behavior. By adding cognitive and motivational factors (perceived autonomy support and intrinsic motivation), we get a better understanding of the interplay between coaches’ leadership behaviors and coachees’ experiences and needs during the coaching process. We demonstrated that transactional and transformational leadership behaviors affect clients’ perceptions and motivation and that these experiences predict coaching success. These are important findings as they provide coaches with the knowledge to create highly effective learning and development environments. To get a profound understanding of the specific features and limits of the different methods (i.e., coaching vs. training), more empirical research is needed that investigates their relative effectiveness and underlying processes.

## Author Contributions

Both authors (SL, ET-M, MM, and EJ) substantially contributed to the conception and the design of the work as well as in the analysis and interpretation of the data. As the first author (SL) prepared the draft, the contributing authors (ET-M, MM, and EJ) reviewed it critically and gave important intellectual input. Both (SL, ET-M, MM, and EJ) worked for the final approval of the version that should be published. Both authors (SL, ET-M, MM, and EJ) are accountable for all aspects of the work in ensuring that questions related to the accuracy or integrity of any part of the work are appropriately investigated and resolved.

## Conflict of Interest Statement

The authors declare that the research was conducted in the absence of any commercial or financial relationships that could be construed as a potential conflict of interest.

The reviewer LP and handling Editor declared their shared affiliation, and the handling Editor states that the process nevertheless met the standards of a fair and objective review.

## Footnotes

1 http://www.coachfederation.org/coachingstudy2012

2 www.coachfederation.org/icfcredentials/core-competencies/.

3 Six weeks after the intervention, participants were sent an online evaluation questionnaire to obtain follow-up data on the long term effects of the intervention program.

4 The procedure and treatments of the participants were approved by the ethical board of the University of Salzburg.

5 In the current study, following variables were measured, but not further analyzed: fear of failure and lack of motivation; self-efficacy; self-regulation; goal clarity; imposter syndrome; trustworthiness; situational intrinsic motivation; self-reflection; pressure and tension; intention to perform a plan; importance of goals and expectations of success; charisma, extra effort, and satisfaction.

6 As participants in the control group received only online questionnaires without interaction with a coach, trainer or contact person, the item was only assessed for the intervention programs individual coaching, self-coaching, and group training. Cronbach’s alpha for the 5-item-scale was α = 0.91.

7 As transactional and transformational leadership behaviors as well as perceived autonomy support refer to behaviors of interaction partners, these mediators were assessed only for the intervention conditions and not for the control condition.

8 There were five cases in the group training condition where goal-attainment ratings at pretreatment (t0) were missing. Missing values were imputed using the expectation-maximization algorithm by the Software Norm (Version 2.03).

9 To test our results on the effectiveness of interventions for significance when self-coaching condition was excluded from the analyses, we further performed group comparisons only between the individual coaching, group training, and control group condition. Results supported our findings for the evaluation criteria and showed that participants’ satisfaction in the individual coaching (*p* < 0.001) and group training condition (*p* = 0.010) was significantly higher compared to control group, whereas individual coaching and group training did marginally significantly differ (*p* = 0.066) in the level of satisfaction, *F*(2, 68) = 9.41, *p* < 0.001, η^2^ = 0.22. For content-related knowledge, scores achieved on the multiple-choice test were significantly higher for participants receiving group training compared to individual coaching, *F*(1,45) = 9.75, *p* = 0.003, η^2^ = 0.18. Goal attainment was significantly higher for the individual coaching condition compared to control group (*p* = 0.028), whereas group training did not significantly differ from the individual coaching (*p* = 0.250) and control group condition (*p* = 0.235), *F*(2,68) = 2.53, *p* = 0.087, η^2^ = 0.07. Regarding state procrastination, individual coaching (*p* = 0.004) reduced procrastination significantly more than no intervention (control group) and group training (*p* = 0.069) reduced procrastination marginally significantly more than no intervention. The individual coaching did not significantly differ from the group training condition in the reduction of procrastination (*p* = 0.207), *F*(2,68) = 4.48, *p* = 0.015, η^2^ = 0.12.
